# Empathy in undergraduate medical students: a multi-center cross-sectional study in China

**DOI:** 10.1186/s12888-023-05350-2

**Published:** 2024-06-04

**Authors:** Runzhi Huang, Zhitong Zhou, Yifan Liu, Min Lin, Meiqiong Gong, Shuyuan Xian, Huabin Yin, Tong Meng, Xiaonan Wang, Yue Wang, Wenfang Chen, Chongyou Zhang, Erbin Du, Xin Liu, Qing Lin, Hongbin Wu, Zongqiang Huang, Jie Zhang, Guoyang Zhang, Shizhao Ji

**Affiliations:** 1 Department of Burns, The First Affiliated Hospital of Naval Medical University, No. 168 Changhai Road, Yangpu District, Shanghai, 200433 China; 2 Tongji University School of Medicine, Shanghai, 200092 China; 3 Shanghai Jiao Tong University School of Medicine, Shanghai, 200025 China; 4 Mental Health Education and Consultation Center, Chongqing Medical University, 61 Daxuecheng Middle Road, Chongqing, 401331 China; 5 Office of Educational Administration, Shanghai University, Shanghai, 200444 China; 6 Department of Orthopedics, Shanghai General Hospital, School of Medicine, Shanghai Jiaotong University, 100 Haining Road, Shanghai, China; 7 Department of Epidemiology and Health Statistics, School of Public Health, Capital Medical University, 10 Xitoutiao, Beijing, 100069 China; 8 Department of Health Statistics, School of Public Health, Air Force Medical University, No.169,Changle West Road, Xi’an, 710032 China; 9 Faculty of Medicine, Jinggangshan University, 28 Xueyuan Road, Ji’An, 343009 China; 10 Basic Medical College, Harbin Medical University, 157 Baojian Road, Heilongjiang, 150081 Heilongjiang China; 11 Frist Clinical Medical College, Mudanjiang Medical University, 66 Tongxiang Street, Mudanjiang, 157011 China; 12 Department of Rheumatology and Immunology, Second Affiliated Hospital of Naval Medical University, Shanghai, China; 13 Department of Human Anatomy, Laboratory of Clinical Applied Anatomy, School of Basic Medical Sciences, Fujian Medical University, 1 Xuefu North Road, Fuzhou, 350122 China; 14 National Centre for Health Professions Education Development, Peking University, Beijing, 100191 China; 15 Institute of Medical Education, Peking University, 5 YiHeYuan Road, Beijing, 100871 China; 16 Department of Orthopedics, The First Affiliated Hospital of Zhengzhou University, 1 Jianshe East Road, Zhengzhou, 450052 China; 17 Department of Gynecology, Shanghai First Maternity and Infant Hospital, Tongji University School of Medicine, 2699 Gaoke West Road, Shanghai, 201204 China; 18 Maastricht University School of Health Professions Education, Maastricht, the Netherlands

**Keywords:** Empathy, Influencing factors, Medical students, Nomogram

## Abstract

**Background:**

Fostering empathy has been continuously emphasized in the global medical education. Empathy is crucial to enhance patient-physician relationships, and is associated with medical students’ academic and clinical performance. However, empathy level of medical students in China and related influencing factors are not clear.

**Methods:**

This was a cross-sectional study among medical students in 11 universities. We used the Jefferson Scale of Empathy Student-version of Chinese version to measure empathy level of medical students. Factors associated with empathy were identified by the univariate and multivariate logistic regression analyses. Based on the variables identified above, the nomogram was established to predict high empathy probability of medical students. Receiver operating characteristic curve, calibration plot and decision curve analysis were used to evaluate the discrimination, calibration and educational utility of the model.

**Results:**

We received 10,901 samples, but a total of 10,576 samples could be used for further analysis (effective response rate of 97.02%). The mean empathy score of undergraduate medical students was 67.38 (standard deviation = 9.39). Six variables including gender, university category, only child or not, self-perception doctor-patient relationship in hospitals, interest of medicine, Kolb learning style showed statistical significance with empathy of medical students (*P* < 0.05). Then, the nomogram was established based on six variables. The validation suggested the nomogram model was well calibrated and had good utility in education, as well as area under the curve of model prediction was 0.65.

**Conclusions:**

We identify factors influencing empathy of undergraduate medical students. Moreover, increasing manifest and hidden curriculums on cultivating empathy of medical students may be needed among medical universities or schools in China.

**Supplementary Information:**

The online version contains supplementary material available at 10.1186/s12888-023-05350-2.

## Introduction

Empathy refers to the ability to recognize and understand the feelings of others [1], is a critical component of human emotional experience and social cognitive ability [2]. The significance of empathy in medical education of medical students is gradually being emphasized among the international medical education. Empathy is a crucial component in satisfactory patient-physician relationship [3]. It has been reported that empathy was associated with academic performance and clinical competence among medical students [3] and patients outcome [4]. Furthermore, higher empathy level was associated with higher self-esteem [5 and lower burnout and distress [6, 7]. Currently, empathy in patient care has been defined as a predominantly cognitive rather than an affective or emotional attribute that involves an ability to understand rather than feel of pain and suffering of patients, a capacity to communicate this understanding, and an intention to help [8].

At present, empathy level has been examined among medical students from multiple countries, such as Brazil [9], Korean [10], Japan [11], Thailand [12], USA [13]. Related factors influencing empathy level of medical students also were widely studied. Numerous studies reported higher empathy scores among female medical students than among male medical students [14, 15], however, no significant difference between gender also was found [16, 17]. Academic year in medical school also was a disputable factor associated with empathy [11, 14, 15, 18], research generally revealed that medical students in the first academic year had higher empathy level [11, 19–21]. Also, most studies found that medical students who preferred a people-oriented specialty had significantly higher empathy scores [9, 13, 20]. Besides, age, parent educational level, race and ethnicity and caring behaviors also were predictors of medical students’ empathy [22, 23]. The empathy of medical students in China has been investigated by some studies already [22, 24, 25], but depths of these studies were not enough, and the sample sizes were limited.

Therefore, it is essential to map the overall landscape of undergraduate medical students’ empathy level in China. We also must understand what factors influence empathy in order to improve it. The main goals of this study are to (i) investigate empathy level of undergraduate medical students in China; (ii) identify factors influencing medical students’ empathy; (iii) construct the nomogram to predict empathy level of medical students based on this multi-center cross-sectional study.

## Materials and methods

### Study design and procedures

The details of study design have been previously published [26]. Briefly, we conducted a cross-sectional study among medical students in 11 universities from 20th, February 2020 to 31rd, March 2020. We selected medical students by stratified cluster random sampling in each university. In each grade, we randomly selected 1 to 2 classes, and all students in each class were selected to complete electronic questionnaire. Data collection was performed by Wenjuanxing (https://www.wjx.cn/). After sorting out all questionnaires collected, the questionnaires with outliers and missing values were eliminated.

### Instrument

The Jefferson Scale of Empathy (JSE) was a widely used instrument to assess empathy level in the context of health professions education and patient care [27]. It has been translated into 56 languages, and used in more than 80 countries [27]. Many studies have used JSE for medical students to assess empathy level of medical students [28–10]. The JSE had three versions, of which the JSE student-version (JSE-S) was mainly used to measure empathy level of medical students [27]. The specific contents of the English version of the JSE-S were displayed in Table S1. It included 20 items answered on a 7-point Likert scale (1 = Strongly Disagree, 7 = Strongly Agree) [27]. Half of the 20 items were positively worded and directly scored, while other half were negatively worded and reverse scored [27]. We used the Chinese version of JSE-S of in this study, and it had good reliability (Cronbach’s α was 0.93 and test–retest reliability was 0.92) and validity (content validity was 0.89 and cumulative variance contribution rate was 57.14%) [30]. The total score ranges of the scale were 20 to 140, and higher values represented higher empathy level.

In the present study, the Kolb’s Learning Style Inventory (LSI) was selected to evaluate predominant learning style of medical students. The LSI was based on Kolb’s experiential learning theory in which learning styles were divided into accommodating, assimilating, converging and diverging with the basis of four learning components, including abstract conceptualization (AC, thinking), concrete experience (CE, feeling), reflective observation (RO, watching), and active experimentation (AE, doing) [31]. Accommodating (CE and AE) in which learners studied by feeling and doing; assimilating (AC and RO) in which learners studied by thinking and watching; converging (AC and AE) in which learners studied by thinking and doing; diverging (CE and RO) in which learners studied by feeling and watching [32].

Nomogram was a graphical statistical prediction model that was widely used to predict the prognosis of diseases, especially in the field of cancers [33]. In this study, therefore, the nomogram was constructed to help educators assess empathy level of medical students.

### Statistical analysis

In this study, data analysis was performed with R version 3.6.1 (Institute for Statistics and Mathematics, Vienna, Austria). Two-sided *P* value < 0.05 was considered as significantly statistical difference. Cronbach’s α value was calculated to assess internal reliability of the scale. Continuous variables were represented as mean ± standard deviation (SD) and categorical variables as number (percentage). Group differences on JSE score were evaluated by two independent sample *t*-test or analysis of variance (ANOVA). Participants were divided into low-level and high-level groups according to the median value of JSE score. The univariate and multivariate logistic regression analyses were used to screen the variables related to empathy of medical students. The nomogram was constructed to predict high empathy probability of medical students. The receiver operating characteristic (ROC) curve was utilized to evaluate predictive accuracy of the nomogram model. The calibration plot was used to assess the consistency between predicted and actual empathy level. The decision curve analysis (DCA) was performed to analyze educational utility of the nomogram model. After sample size estimation, 2700 participants were sufficient to detect significantly statistical difference.

## Results

### Sample characteristics

**Fig. 1:**
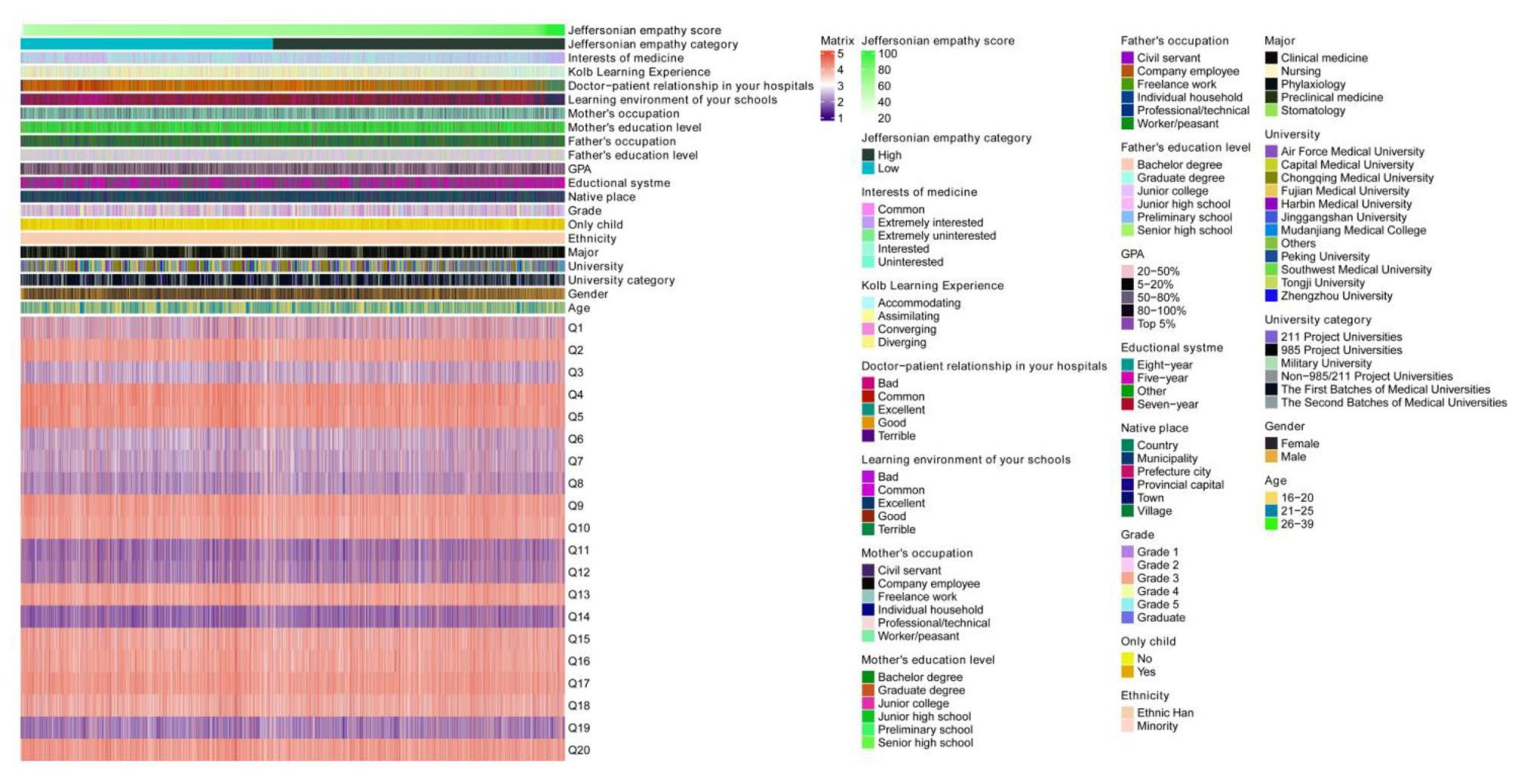
Heatmap of JSE score. JSE, Jefferson Scale of Empathy; GPA, grade point average

Data were collected from 10,901 undergraduate medical students. After eliminating invalid questionnaires, a total of 10,576 samples were included in the statistical analysis, with an effective response rate of 97.02%. In our study, the scale presented good internal consistency (Cronbach’s α = 0.87). The mean empathy score of medical students was 67.38 (SD = 9.39). The age of medical students was mainly concentrated on 16–25 years (98.79%). Compared with this age range, students in 26–39 years had higher empathy scores (*P* = 0.002). Males had higher empathy scores than females (*P* < 0.001). Regarding the university category, students in the First Batches of Medical Universities had higher empathy scores, however, in the Project 985 Universities had lower empathy scores (*P* < 0.001). Students who were only child in the family had higher empathy scores (*P* < 0.001). Students with high educational level of parents showed higher empathy level (*P* < 0.05). The better the students’ self-perception about the current learning environment of school and doctor − patient relationship of hospitals, the higher the empathy scores of medical students (*P* < 0.001). In addition, students who were more interested in medicine had higher empathy level (*P* < 0.001), and who took accommodating as a main learning style had higher empathy scores (*P* < 0.001) (Table 1; Fig. 1).

**Table 1:** Baseline characteristics and JSE score of 10,576 subjects

Variables	Number (percentage)	JSE score (mean ± SD)	*P*-value
**Age**			0.002*
16–20	5715 (54.04)	67.62 ± 9.59	
21–25	4733 (44.75)	67.05 ± 9.11	
26–39	128 (1.21)	68.87 ± 10.34	
**Gender**			< 0.001*
Male	4205 (39.76)	68.90 ± 11.27	
Female	6371 (60.24)	66.37 ± 7.75	
**University category**			< 0.001*
Non − 985/211 Project Universities	720 (6.81)	68.29 ± 10.11	
211 Project Universities	692 (6.54)	68.32 ± 10.10	
985 Project Universities	853 (8.07)	65.66 ± 8.01	
Military University	526 (4.97)	66.92 ± 8.68	
The First Batches of Medical Universities	6473 (61.20)	69.91 ± 12.08	
The Second Batches of Medical Universities	1312 (12.41)	67.05 ± 8.89	
**Major**			0.664
Clinical medicine	8371 (79.15)	67.38 ± 9.50	
Nursing	567 (5.36)	66.86 ± 7.51	
Phylaxiology	689 (6.52)	67.59 ± 9.38	
Preclinical medicine	652 (6.16)	67.50 ± 9.55	
Stomatology	297 (2.81)	67.62 ± 9.39	
**Ethnicity**			0.790
Ethnic Han	9893 (93.54)	67.37 ± 9.41	
Minority	683 (6.46)	67.47 ± 9.17	
**Only child**			< 0.001*
No	5977 (56.51)	66.94 ± 8.71	
Yes	4599 (43.49)	67.94 ± 10.18	
**Grade**			< 0.001*
Grade 1	3722 (35.19)	67.87 ± 9.60	
Grade 2	1986 (18.78)	67.58 ± 9.77	
Grade 3	1639 (15.50)	66.67 ± 8.85	
Grade 4	1843 (17.42)	66.74 ± 9.06	
Grade 5	1254 (11.86)	67.56 ± 9.41	
Graduate	132 (1.25)	66.45 ± 7.52	
**Native place**			< 0.001*
Village	2366 (22.37)	66.74 ± 8.319	
Town	1131 (10.69)	66.78 ± 8.32	
Prefecture city	1974 (18.67)	68.20 ± 10.14	
Provincial capital	1088 (10.29)	67.67 ± 9.88	
Municipality	1484 (14.03)	67.19 ± 9.89	
Country	2533 (23.95)	67.59 ± 9.62	
**Educational system**			< 0.001*
Five − year (n = 7376)	7376 (69.74)	67.69 ± 9.84	
Seven − year (n = 280)	280 (2.65)	66.45 ± 9.53	
Eight − year (n = 1281)	1281 (12.11)	66.47 ± 8.43	
Other (n = 1639)	1639 (15.50)	66.82 ± 7.81	
**GPA**			0.831
Top 5%	758 (7.17)	67.73 ± 10.70	
5–20%	2431 (22.99)	67.33 ± 8.88	
20–50%	3744 (35.40)	67.36 ± 9.18	
50–80%	2640 (24.96)	67.30 ± 8.90	
80–100%	1003 (9.48)	67.49 ± 11.39	
**Father’s education level**			0.011
Preliminary school	1769 (16.73)	66.73 ± 9.01	
Junior high school	3721 (35.18)	67.40 ± 8.99	
Senior high school	2514 (23.77)	67.60 ± 10.03	
Junior college	1104 (10.44)	67.16 ± 9.07	
Graduate degree	233 (2.20)	67.80 ± 8.76	
Bachelor degree	1235 (11.68)	67.90 ± 10.12	
**Father’s occupation**			0.504
Civil servant	1032 (9.76)	67.78 ± 10.14	
Company employee	1057 (9.99)	67.20 ± 9.77	
Freelance work	2062 (19.50)	67.57 ± 9.39	
Individual household	1056 (9.98)	67.40 ± 9.34	
Professional/technical	1103 (10.43)	67.39 ± 9.30	
Worker/peasant	4266 (40.34)	67.22 ± 9.14	
**Mother’s education level**			< 0.001*
Preliminary school	3126 (29.56)	66.61 ± 8.79	
Junior high school	3241 (30.65)	67.60 ± 9.16	
Senior high school	2159 (20.41)	67.64 ± 9.77	
Junior college	977 (9.24)	67.94 ± 10.03	
Graduate degree	163 (1.54)	67.55 ± 10.40	
Bachelor degree	910 (8.60)	67.97 ± 10.23	
**Mother’s occupation**			0.086
Civil servant	599 (5.66)	67.42 ± 10.26	
Company employee	1206 (11.40)	67.12 ± 9.67	
Freelance work	2816 (26.63)	67.29 ± 9.09	
Individual household	770 (7.28)	67.39 ± 9.60	
Professional/technical	1308 (12.37)	68.11 ± 10.07	
Worker/peasant	3877 (36.66)	67.27 ± 19.10	
**Learning environment of your schools**			< 0.001*
Terrible	60 (0.57)	65.47 ± 19.49	
Bad	116 (1.10)	64.09 ± 11.09	
Common	2210 (20.89)	65.82 ± 7.85	
Good	5898 (55.77)	66.52 ± 7.55	
Excellent	2292 (21.67)	71.31 ± 12.83	
**Doctor − patient relationship in your hospitals**			< 0.001*
Terrible	45 (0.42)	63.71 ± 22.07	
Bad	117 (1.11)	64.54 ± 8.80	
Common	2753 (26.03)	65.67 ± 7.94	
Good	6009 (56.82)	66.65 ± 7.47	
Excellent	1652 (15.62)	73.16 ± 14.05	
**Kolb learning style**			< 0.001*
Accommodating	3572 (33.77)	69.38 ± 11.23	
Assimilating	3119 (29.49)	65.59 ± 6.92	
Converging	1734 (16.40)	66.74 ± 8.38	
Diverging	2151 (20.34)	67.15 ± 19.32	
**Interests of medicine**			< 0.001*
Extremely uninterested	65 (0.62)	61.60 ± 15.72	
Uninterested	161 (1.52)	63.76 ± 7.93	
Common	2599 (24.57)	65.33 ± 7.39	
Interested	5970 (56.45)	66.75 ± 7.60	
Extremely interested	1781 (16.84)	72.99 ± 13.96	

JSE, Jefferson Scale of Empathy; SD, standard deviation; GPA, grade point average. ∗ *P* < 0.05

### Factors associated with medical students’ empathy

We performed the univariate logistic regression analysis, and six variables including gender, university category, only child or not, self-perception doctor-patient relationship in hospitals, interest of medicine, Kolb learning style were associated with empathy (*P* < 0.05) (Table S2). Then, the multivariate logistic regression model was constructed based on six variables. In the model, we found that males had higher empathy level compared with females (OR, 1.30; 95%CI, 1.20–1.41; *P* < 0.001). Compared to the “Project 211” universities, medical students in the “Project 985” universities were more likely to have lower empathy (OR, 0.61; 95%CI, 0.49–0.75; *P* < 0.001). Only child in the family had higher empathy scores (OR, 1.11; 95%CI, 1.02–1.20; *P* = 0.014). Medical student who reported better doctor-patient relationship in hospitals presented higher empathy level [common (OR, 1.52; 95%CI, 1.03–2.26; *P* = 0.038); good (OR, 1.77; 95%CI, 1.21–2.63; *P* = 0.004); excellent (OR, 2.90; 95%CI, 1.94–4.38; *P* < 0.001)]. Moreover, medical students more interested in medicine were more likely to have higher empathy [interested (OR, 1.45; 95%CI, 1.32–1.60; *P* < 0.001); extremely interested (OR, 2.30; 95%CI, 1.99–2.67; *P* < 0.001)]. Students who preferred accommodating learning style had higher empathy, however, assimilating learning style had lower empathy (OR, 0.66; 95%CI, 0.59–0.73; *P* < 0.001) (Table 2).

**Table 2:** Multivariate logistic regression analysis of empathy

Variables	OR (95% CI)	*P*-value
**Gender**		
Female	1.00 (reference)	
male	1.30 (1.20–1.41)	< 0.001*
**University category**		
211 Project Universities	1.00 (reference)	
985 Project Universities Military University Non-985/211 Project Universities The First Batches of Medical Universities The Second Batches of Medical Universities	0.61 (0.49–0.75) 0.73 (0.57–0.92) 0.89 (0.72–1.11) 0.77 (0.66–0.91) 0.99 (0.82–1.21)	< 0.001* 0.008* 0.289 0.002* 0.932
**Only child**		
No	1.00 (reference)	
Yes	1.11 (1.02–1.20)	0.014*
**Doctor patient relationship in your hospitals**		
Bad	1.00 (reference)	
Terrible Common Good Excellent	1.02 (0.48–2.16) 1.52 (1.03–2.26) 1.77 (1.21–2.63) 2.90 (1.94–4.38)	0.953 0.038* 0.004* < 0.001*
**Interests of medicine**		
Common	1.00 (reference)	
Extremely uninterested Uninterested Interested Extremely interested	0.72 (0.41–1.22) 0.76 (0.54–1.06) 1.45 (1.32–1.60) 2.30 (1.99–2.67)	0.227 0.105 < 0.001* < 0.001*
**Kolb learning style**		
Accommodating	1.00 (reference)	
Assimilating Converging Diverging	0.66 (0.59–0.73) 0.78 (0.70–0.88) 0.80 (0.72–0.90)	< 0.001* < 0.001* < 0.001*

OR, odds ratio; CI, confidence interval. ^∗^*P* < 0.05

### The nomogram prediction of empathy and validation

**Fig. 2:**
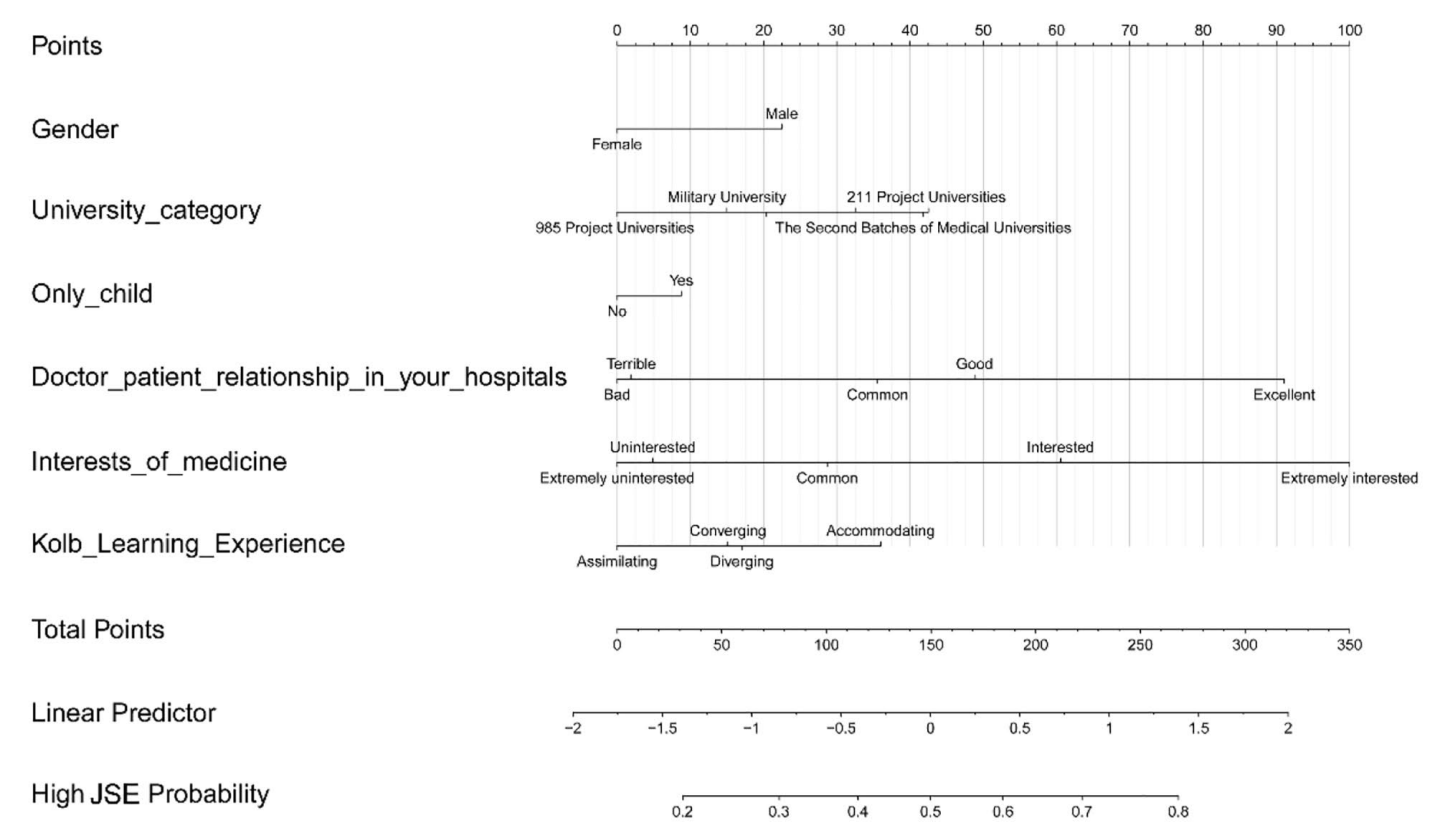
Nomogram prediction of medical students’ empathy level. JSE, Jefferson Scale of Empathy

**Fig. 3:**
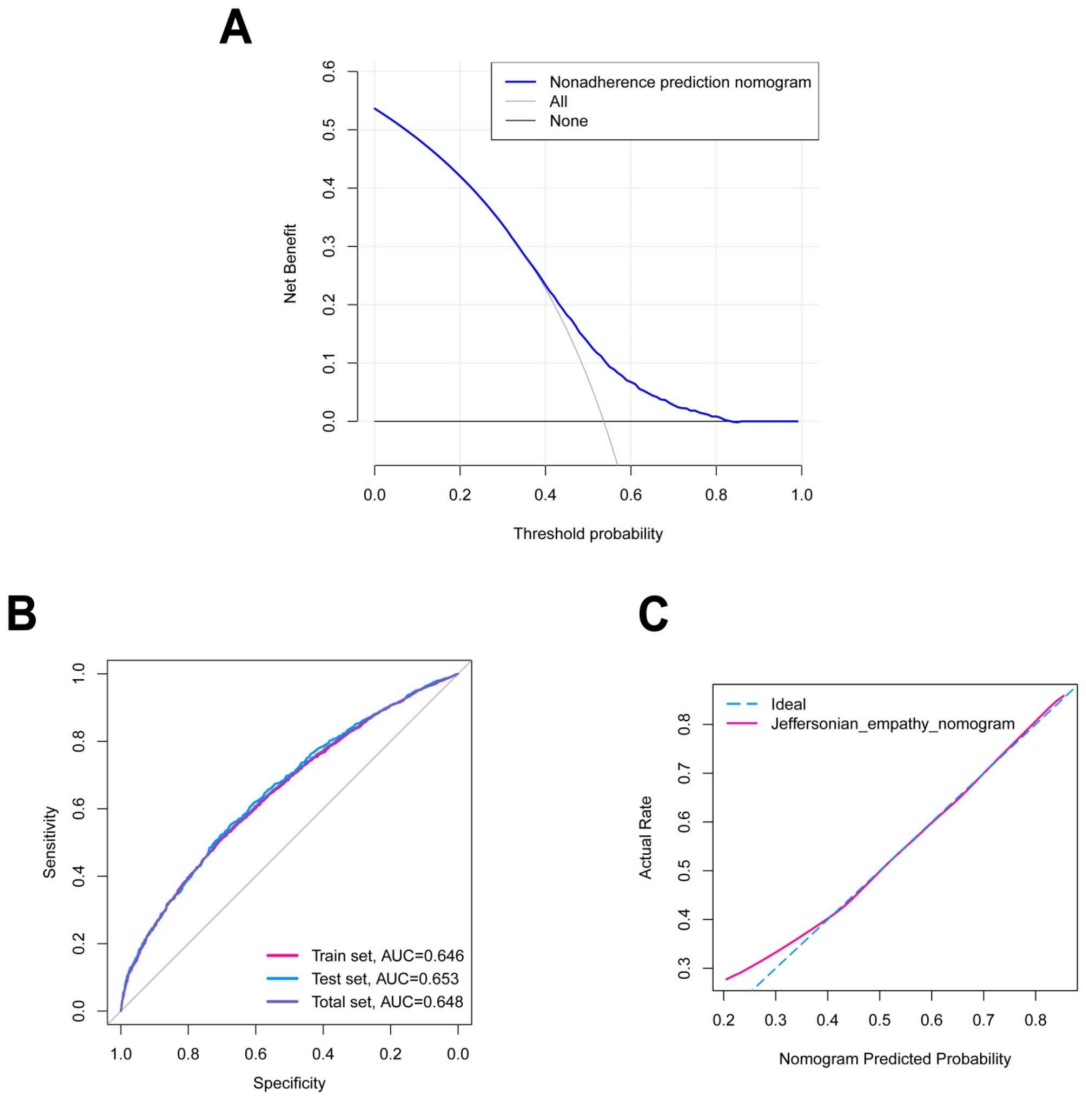
DCA (A), ROC curve (B) and calibration (C) of the nomogram. DCA was used to assess educational utility of the nomogram, and DCA suggested that the nomogram had good utility. Predictive accuracy of the model was analyzed by ROC curve, and ROC curve showed that the model had general predictive accuracy (Total set AUC = 0.648, Train set AUC = 0.646, Test set AUC = 0.653). Calibration was used to evaluate how close the nomogram estimated risk was to the actual risk, and the calibration pot indicated the nomogram were well-calibrated. DCA, decision curve analysis; ROC, receiver operating characteristic; AUC, area under the curve

Based on the multivariate logistic regression model, we established the nomogram to predict the probability of high empathy scores (Fig. 2). Firstly, we evaluated educational utility of the model using DCA, and Fig. 3A showed the model had good utility. Additionally, the area under the curve (AUC) of model prediction was 0.65 (Fig. 3B). Furthermore, Fig. 3C showed the calibration plot was close to standard plot, which indicated that the model had good prediction accordance.

## Discussion

In this study, we mainly aimed to determine relevant factors affecting empathy level of medical students. The present study showed that six variables were associated with medical students’ empathy, including gender, university category, only child or not, self-perception about doctor-patient relationship in hospitals, interest of medicine, Kolb learning style. Furthermore, the nomogram model that predicted empathy level of medical student showed good predictive consistency and educational utility. But the model only presented general discriminative ability (AUC = 0.65), which indicated that the model might not include other important factors that affected empathy, so it was necessary to explore more factors that influenced empathy, and then established a more accurate prediction model.

The mean empathy score of medical students was 67.38 in our study, which was lower than the result of other countries (mean score ranging from 91 to 120) [10, 29, 34–20] and China (mean score of 104 and 109) [22, 36]. Difference in empathy level from other countries might be related to cultural differences, such as social norms, religious belief and teaching mode [22, 36, 37]. Medical education in China starts with undergraduate period, students can directly apply for medical majors in universities after graduating from high school, while starts after completing undergraduate studies in the United States. Undergraduate graduates have already had some social and life experiences during their college period, social science is conducive to fostering individual empathy, which may lead to relatively low level of empathy among medical students in China [36]. Besides, studies in our country were concentrated on one university or one region, which cannot represent empathy level of Chinese medical students. The 11 universities were located at different regions of our country, which could better represent empathy level among medical students in China.

In our study, there was a significant difference of empathy scores between gender, males outscored females. Study reported that female medical students had higher distress [38]. The distress could significantly decline empathy level [39], so females might show lower empathy level than males. The finding was controversial with other studies [10, 20, 40, 41], So more large-sample studies should be carried out to explore the gender difference and related reasons in medical students’ empathy. The difference in empathy between universities might be attributed to different educational patterns, concepts, and requirements for students in the medical school of each university. In China, “Project 985” universities are key universities in China, aiming to promote the development of high-quality education [42]. Students who were from these universities had the lowest empathy scores in this study, these students might face higher learning pressure that was an important factor of empathy decrease [39]. In addition, elitist thinking and belonging to an elitist group might also affect empathy level [39].

Medical students with only child presented higher level of empathy in the present study. Previous study found an inverse correlation between the number of brother or sister and empathy [21]. Only child could obtain more parent’ concern, attention and encouragement, helping child develop higher resilience level that was conductive to keep more stable mental health and reduce anxiety and depression [43]. Non-only child more perceived stress and study-related life dissatisfaction than only child [44]. Good psychological status was a protective factor of empathy level [12]. A better doctor-patient relationship in hospital will have a more positive impact on empathy of medical students. An appropriate role models in hospitals also had a positive influence on empathy [41]. Therefore, improving the relationship between doctors and patients and setting good role models might be helpful to improve empathy of medical students. Students who were more interested in medicine had higher empathy scores, which was consistent with other studies [20, 40, 45]. Interest is a driving factor of learning motivation. Thus, in the process of medical education, medical schools should also pay attention to cultivating medical students’ interest in medicine. Accommodating is a learning style by feeling and doing and accommodator preferred learning from “hands-on” experience [46]. The study reported that increasing hands-on experiences could promote development of empathy [47]. Clinical practice phase was a key factor of empathy decline [39]. Moreover, medical students who pursued people-oriented specialties had higher empathy level than technology-oriented Specialties [41].

The change of empathy level during academic years was still controversial [1]. But medical students’ empathy could be enhanced and sustained in medical school by targeting educational programs [48]. A number of methods have been described to enhance empathy in medical education, such as communication skills training, communicating more with patients, audio-or video-taping of encounters with patients, exposure to role models, role playing (aging game), shadowing a patient (patient navigator), hospitalization experiences, studying literature and arts, improving narrative skills, theatrical performances, and the Balint method [47–50]. It was essential that empathy training was incorporated in the early stage of medical education and ensuring its sustained development [51].

There are some implications in the present study. First, medical schools should attach importance to clinical practice and increase communication opportunities between medical students and patients in practice. Besides, we suggest that empathy should be included in the assessment of internship performance. Importantly, Chinese medical schools should increase the teaching of empathy among medical students in the curriculum setting, and adopt some methods to maintain their empathy level.

To our best knowledge, this is the first study to predict empathy level of medical students by constructing nomogram in China, Furthermore, the 11 universities are in multiple regions of China, so the research results are more representative and applicable. Nevertheless, there are some limitations in this study. Firstly, this is a cross-sectional study, influence of cohort effects cannot be completely dismissed. Thus, longitudinal studies of verifying the findings are warranted. Secondly, we use a self-reporting scale, so there are possible differences between self-report and actual contents. Thirdly, we do not comprehensively collect characteristic information of the participants and universities, which will lead to the existence of residual confounding. Fourthly, current findings are based on Chinese population, therefore, they are not be applied to other ethnic population.

## Conclusion

The present study indicated that gender, university category, only child or not, self-perception about doctor-patient relationship in hospitals, interest of medicine, Kolb learning style were predictors of empathy of medical students. More large-sample studies or qualitative research should be conducted to investigate the influencing factors of empathy of medical students, further to facilitate medical students’ empathy. We suggest that increasing curriculums on cultivating empathy of medical students, paying attention to clinical practice and including empathy in practice assessment, cultivating the ability to communicate and handle doctor-patient relationship of medical students may be needed in Chinese medical schools.

### Electronic supplementary material

Below is the link to the electronic supplementary material.

Supplementary Material 1

## Data Availability

The datasets used and/or analysed during the current study are available from the corresponding author on reasonable request.
