# Supplementary material for: Empathy in undergraduate medical students: a multi-center cross-sectional study in China
Source: BMC Psychiatry. 2024 Jun 4;24:414. doi: 10.1186/s12888-023-05350-2 (PMC11151548; doi:10.1186/s12888-023-05350-2)
Supplement: Supplementary file 1 — Supplementary Material 1 [file 12888_2023_5350_MOESM1_ESM.docx]

**Supplementary materials**

| Scale items |
| --- |
| 1. A physician who is able to view things from another person’s perspective can render better care. |
| 2. Physicians’ sense of humor contributes to a better clinical outcome. |
| 3. Physicians’ understanding of their patients’ feelings and the feelings of their patients’ families is a positive treatment factor. |
| 4. For more effective treatment, physicians must be attentive to their patients’ personal experiences. |
| 5. Understanding body language is as important as verbal communication in physician-patient relationships. |
| 6. Empathy is an important therapeutic factor in medical treatment. |
| 7. Patients feel better when their feelings are understood by their physicians. |
| 8. Physicians’ demonstration of understanding their patients’ emotions is an important factor in interviewing and history taking. |
| 9. Willingness to imagine oneself in another person’s place contributes to providing quality care. |
| 10. Patients’ illness can be cured only by medical treatment; physicians’ affectional ties with their patients do not have a significant place in this endeavor. |
| 11. What is going on in a patient’s mind can often be expressed by nonverbal cues such as facial expressions or body language that must be carefully observed by physicians. |
| 12. A patient who feels understood can experience a sense of validation that is therapeutic in its own right. |
| 13. One important component of the successful physician-patient relationship is the physician’s ability to understand the emotional status of his or her patients and their families. |
| 14. It is as important to ask patients about what is happening in their lives as it is to ask about their physical complaints. |
| 15. It is acceptable for a physician to be touched by intense emotional relationships between patients and their families. |
| 16. Reading nonmedical literature and enjoying the arts can enhance physicians’ ability to render better care. |
| 17. Because people are different, it is almost impossible for physicians to see things from their patients’ perspectives. |
| 18. Emotion has no place in the treatment of medical illness. |
| 19. Empathy is a therapeutic skill without which the physician’s success will be limited. |
| 20. The best way to take care of a patient is to think like a patient. |

Table S1 The Jefferson Scale of Empathy Student-version

Table S2 Univariate logistic regression analysis of empathy

| Variables | *P*-value |
| --- | --- |
| Age | 0.704 |
| Gender | <0.001* |
| University category | <0.001* |
| University | 0.909 |
| Major | 0.869 |
| Ethnicity | 0.396 |
| Only child | <0.001* |
| Grade | 0.097 |
| Native place | 0.400 |
| Educational system | 0.149 |
| GPA | 0.594 |
| Father education level | 0.954 |
| Father occupation | 0.264 |
| Mother education level | 0.399 |
| Mother occupation | 0.475 |
| Learning environment of your schools | 0.420 |
| Doctor patient relationship in your hospitals | 0.028* |
| Interests of medicine | <0.001* |
| Kolb learning style | <0.001* |
